# Thalamic deep brain stimulation for postural tremor caused by hyperthermia-induced cerebellar dysfunction: A case report

**DOI:** 10.1016/j.ensci.2024.100536

**Published:** 2024-11-17

**Authors:** Mitsuyoshi Tamura, Shigeki Hirano, Yoshihisa Kitayama, Marie Morooka, Tomoki Suichi, Kazumoto Shibuya, Yoshinori Higuchi, Satoshi Kuwabara

**Affiliations:** a Department of Neurology, Graduate School of Medicine, Chiba University, Chiba, Japan; b Department of Neurological Surgery, Graduate School of Medicine, Chiba University, Chiba, Japan

**Keywords:** Hyperthermia, Cerebellar dysfunction, Postural tremor, Deep brain stimulation

## Abstract

**Background:**

The efficacy of deep brain stimulation (DBS) in treating tremor symptoms in cerebellar disorders remains unclear.

**Case presentation:**

A 47-year-old woman presented with neck and arm tremor and ataxic speech/gait after four days of >40 °C fever due to septic shock attributed to lithiasis-pyelonephritis. Left ventral intermediate nucleus thalamus DBS alleviated contralateral postural arm tremor, although the action tremor and terminal oscillation remained unchanged.

**Discussion:**

To our knowledge, this is the first report of thalamic DBS for hyperthermia-induced cerebellar dysfunction. Patients with postural tremor resulting from cerebellar damage can benefit from thalamic DBS, leading to improved activities of daily living.

## Highlights

• Hyperthermia cerebellar dysfunction is a condition in which the cerebellar Purkinje cells degenerate due to hyperthermia.
• Cerebellar tremor is a postural and action tremor that is caused by a disturbance of the cerebello-thalamo-cortical pathway.
• Thalamic deep brain stimulation was effective for cerebellar-postural tremor in hyperthermia cerebellar dysfunction.

## 1 Introduction

Cerebellar disorders encompass a wide spectrum of conditions arising from diverse sources, including neurodegeneration, inflammation, heat stroke, and alcohol intoxication, among others [1]. Cerebellar tremor, one of the major symptoms in patients with cerebellar dysfunction, is devastating and significantly interferes with activities of daily living; however, treatment and management strategies remain poorly characterized. Hence, its pathophysiology requires elucidation [2].

Deep brain stimulation (DBS) is a well-established treatment for tremor disorders; however, evidence to support its use for cerebellar disorders is insufficient [3]. To the best of our knowledge, no patients with hyperthermia-induced cerebellar dysfunction who have undergone DBS have been reported to date. Here, we report a patient with hyperthermia-induced cerebellar dysfunction who underwent unilateral thalamic (ventralis intermedius nucleus, Vim) DBS for tremor symptoms.

### 1.1 Case presentation

A 47-year-old right-handed Japanese woman with a medical history of a right arm injury in childhood, resulting in retained hand contracture, was admitted to the emergency room with impaired consciousness and high fever since the previous day. She was diagnosed with septic shock caused by right lithiasis pyelonephritis, and was started on mechanical ventilatory support in the intensive care unit. A fever exceeding 40 °C was sustained for four days after admission. Nephrostomy and antibiotics led to resolution of the pyelonephritis, and the patient no longer required ventilatory support by the fifth day of admission. However, she exhibited tremors in the neck and both upper limbs and later noticed that she had difficulty walking due to ataxia. Daily doses of clonazepam (1.5 mg) and zonisamide (100 mg) were prescribed in an attempt to manage tremor symptoms, but with minimal effect (Video 1, Segment 1). Four months later, the patient was referred to our hospital for further examination.

**Fig. 1:**
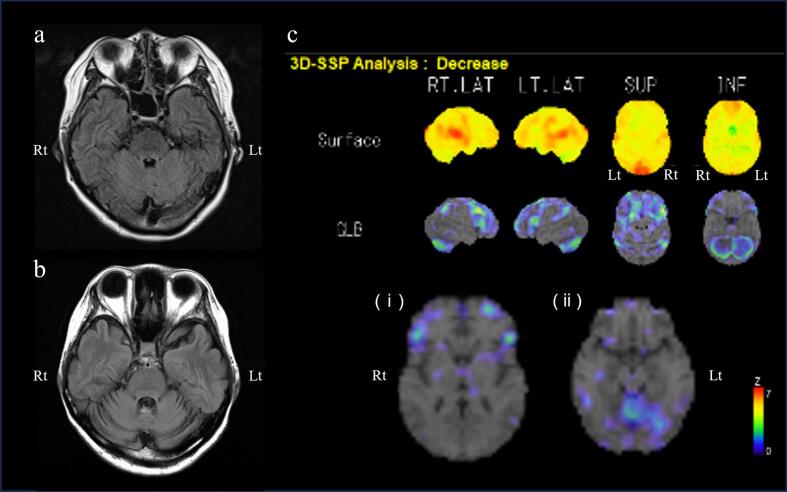
Temporal changes in brain magnetic resonance and perfusion images before surgery. (a) Transverse slices of fluid-attenuated inversion recovery (FLAIR) images during the acute phase of hyperthermia. (b) Transverse FLAIR images taken at nine months after onset, showing progressive atrophy of the bilateral superior cerebellar peduncles and the cerebellum. (c) Three-dimensional stereotactic surface projection analyses of ^123^I-iodoamphetamine single-photon emission computed tomography images, scanned nine months after onset. Statistical parametric image of reduced regional perfusion shown in rainbow color (Z score) compared to the healthy individual database, showing marked hypoperfusion in the bilateral cerebellum and mild hypoperfusion in the bilateral frontal lobes, but no abnormalities in the (i) basal ganglia and (ii) brainstem in the transverse slice. Rt: right, Lt: left.

On admission, she was administered 6 mg clonazepam daily, which induced daytime drowsiness. She had bilateral horizontal gaze-directional nystagmus and hypermetric eye movements. Her speech was highly ataxic, with no palatal tremors. Severe left-predominant bilateral motor decomposition and dysmetria on the finger-to-nose test were noted. Terminal oscillation and postural and action tremors with a frequency of 3–5 Hz were observed in both upper limbs (Video 1, Segment 1), but no tremors occurred at rest. She was able to rise from a chair with light assistance, but experienced severe difficulty walking due to truncal ataxia. Her scores on the Fahn–Tolosa–Martin Tremor Rating Scale (TRS) was 72/144, International Cooperative Ataxia Rating Scale (ICARS) was 58/100, and Scale for the Assessment and Rating of Ataxia (SARA) 23.5/40 (Table 1). Brain magnetic resonance imaging revealed atrophy and fluid-attenuated inversion recovery with high-intensity signal changes in the bilateral cerebellum and superior cerebellar peduncles, which progressed over time (Fig. 1a, b). ^123^I-iodoamphetamine (^123^I-IMP) single-photon emission computed tomography indicated marked hypoperfusion in the bilateral cerebellum and frontal lobes, while the basal ganglia and brainstem showed normal perfusion (Fig. 1c).

**Table 1:** Clinical assessment scores before and after left thalamic deep brain stimulation surgery.

	before surgery	two years after surgery	two years after surgery
		DBS on	DBS off
medication	clonazepam 6 mg	clonazepam 0.5 mg	clonazepam 0.5 mg
TRS	72	57	64
TRS on U/E	right: 3, left: 6	right: 2, left: 3	right: 3, left: 5
SARA	23.5	15	15
ICARS	58	35	35
Barthel index	55	80	
Feeding	5	5	–
Bathing	0	0	–
Grooming	5	5	–
Dressing	5	5	–
Bowels	10	10	–
Bladder	10	10	–
Toilet	5	10	–
Transfers	10	15	–
Mobility	5	10	–
Stairs	0	10	–

DBS, deep brain stimulation; ICARS, International Cooperative Ataxia Rating Scale; SARA, Scale for the Assessment and Rating of Ataxia; TRS, Fahn-Tolosa-Martin Tremor Rating Scale.

The patient was diagnosed with hyperthermia-induced cerebellar dysfunction. Tremors were treated with daily doses of clonazepam (6 mg) and valproic acid (600 mg), but were intolerable because of drowsiness, which significantly affected daily life (Video 1, Segment 2). Levodopa was also ineffective. Beta-blocker was not prescribed because of hypotension, primidone was not prescribed because of ineffective zonisamide, and 4-aminopyridine was not commercially available in Japan. Nevertheless, the patient remained motivated to undergo treatment, due to the serious consequences of hand tremors. As the existing literature suggests the partial efficacy of DBS in addressing cerebellar tremors [3], a DBS procedure was considered, and a comprehensive discussion and meticulous deliberation were made involving the patient, her family, professional neurologists, and neurosurgeons. After obtaining written informed consent, DBS electrodes were implanted into the left Vim nucleus of thalamus [Twist lead (0.5-mm interval), Vercise Gevia™, Boston Scientific] with the aim of improving tremor symptoms in the dominant hand. Because of the potential refractoriness, simultaneous stimulation of both the Vim and posterior subthalamic area was initially planned, but required modification because of the difficulty in their anatomical location. Instead, the electrode was positioned slightly deeper in the ventral portion of the left Vim nucleus to ensure that its tip resided within the zona incerta.

Under stimulation (140 μs, 185 Hz, 3.6 mA), the postural tremor of the right arm markedly improved (Video 1, Segment 3). Manipulation of objects at a distance from the body, such as turning pages while reading a book, was relatively smooth. However, no obvious changes were observed in terms of action tremor, terminal oscillation, or motor decomposition. In other words, there were little or no improvements in actions that required precise target attainment and concentration, such as bringing a meal to the mouth or pouring water into a glass. Over two years follow up, during which she took clonazepam (0.5 mg/day) and rehabilitation, her TRS, ICARS, and SARA scores were 57/144, 35/100, and 15/40, respectively (Table 1). Activities of daily living were assessed using the Barthel Index which was 55/100 pre-DBS (on initial admission) and improved to 80/100 two years post-DBS (Table 1), attributable to improvement in the reduction in tremor and in her walking ability.

Additionally, on two years after surgery, symptoms were also evaluated with DBS-on state and DBS-off state (Video 1, Segment 4 and 5). Postural tremor was worse in both hands with DBS off. TRS upper extremity sub scores of right 2 (on) and 3 (off), left 3 (on) and 5 (off). Limb ataxia and terminal oscillation remained unchanged between DBS on and off (Table 1).

## 2 Discussion

The tremor in the present case was a postural and action tremor of 3–5 Hz without rest tremor. Cerebellar tremor is defined as an irregular, high-amplitude, postural action tremor without rest tremor [4], which was consistent with the tremor in the present case. The pathophysiology and functional anatomy of cerebellar tremor remain elusive, but may be ascribed by altered functional connectivity (malfunction and compensation) inside and outside the cerebellum. In two major cerebellar efferent pathways: the cerebello-thalamo-cortical pathway (cerebellar cortex-dentate nucleus-superior cerebellar peduncle-red nucleus-ventral lateral nucleus-cerebral cortex) and the dentato-rubro-olivary pathway (also known as Guillain-Mollaret triangle), the former pathway is involved in cerebellar tremor, and the latter pathway in Holmes tremor [[5], [6], [7]]. Although the two pathways share a route from the dentate nucleus to the superior cerebellar peduncle, no concrete conclusions have yet been drawn regarding the location of the tremor hub center or the type of tremor that corresponds to this region. The most important finding in our patient was the improvement in postural tremor after Vim DBS. This could provide a basis for the possible mechanism of cerebellar tremor, in which the postural tremor component may account for the influence of the Vim nucleus. In contrast, the unchanged action tremor (strongly influenced by intention) and terminal oscillation indicate that the intention component can probably be explained by a disturbance in other pathways, such as those of the middle and/or inferior cerebellar peduncles. However, several reports have shown the efficacy of Vim DBS in patients with Holmes tremor caused by damage to the Guillain-Mollaret triangle, a pathway that does not pass through the Vim [8]. Taken together, these results indicate that cerebellar tremor and Holmes tremor may, in part, have common underlying pathophysiologies, and from the view of the present case, the circuit responsible for postural tremor induced by cerebellar dysfunction may be mediated by the Vim nucleus, while the lesion and circuit responsible for intention tremor may differ from those two major cerebellar pathways.

Another value of the present case is that it is the first in which DBS was performed to treat hyperthermia-induced cerebellar dysfunction. This condition is known as a residual symptom of heat stroke, neuroleptic malignant syndromes or infections, and some patients exhibit long-term cerebellar dysfunction [9].

Targets of DBS in cerebellar disorders vary, and may include the Vim, PSA, or globus pallidus internus (GPi), but all have been reported to achieve an improvement of 50–70 % in TRS [3], as in our patient. Regarding the effectiveness of unilateral DBS in this case of bilateral postural tremor, previous reports indicated that there are some cases in which unilateral Vim DBS was effective bilaterally [10]. Since ablative therapy against tremor symptoms in cerebellar dysfunction, such as focused ultrasound surgery, is partially effective, it may play a role in managing tremor symptoms, notably the Vim nucleus targeted against postural tremors, in ataxic patients. The efficacy of DBS may differ depending on the underlying disease, and further studies are required to draw conclusions.

## 3 Conclusion

Herein, we report a case of hyperthermia-induced cerebellar dysfunction in which Vim DBS was effective at treating contralateral and lesser ipsilateral postural tremors. Postural tremors caused by cerebellar dysfunction may involve the Vim pathway, whereas intention tremors may involve other pathways. Future studies are needed to draw conclusions regarding the efficacy of brain surgical procedures for cerebellar dysfunction; however, patients may benefit from these interventions.

The following are the supplementary data related to this article.

Supplementary Video 1 Videos of postural tremors in the current patient with hyperthermia-induced cerebellar dysfunction.
Segment 1: Four months after onset, the patient was taking clonazepam 1.5 mg and zonisamide 100 mg per day, which was refractory to control postural tremor.
Segment 2: Ten months after onset, the patient took 6 mg of clonazepam and 600 mg of valproic acid per day, which was effective for postural tremor but induced prominent drowsiness. Intention tremor was observed in finger-to-nose test.
Segment 3: After deep brain stimulation (DBS) surgery, the postural tremors improved, and the patient took 0.5 mg of clonazepam per day.
Segment 4: Two years after surgery, symptoms were evaluated with DBS-off state. Postural tremor was worse in her both arms when DBS was off. Remarkable intention tremor remained in finger-to-nose test.
Segment 5: Two years after surgery, symptoms were evaluated with DBS-on state. Postural tremor was improved when DBS was on. However, intention tremor in finger-to-nose test remained unchanged between DBS-on state and DBS-off state. Supplementary Video 1

## Funding

This study received no specific grants from any funding agencies in the public, commercial, or not-for-profit sectors.

## Statement of informed consent

The author (MT) explained to the patient that medical information and data would be presented in anonymized form, and obtained the patient's consent. The patient's written consent was obtained during a follow-up consultation. The written consent was consigned in the patient's medical record.

## CRediT authorship contribution statement

**Mitsuyoshi Tamura:** Writing – original draft, Resources, Project administration, Investigation, Data curation, Conceptualization. **Shigeki Hirano:** Writing – review & editing, Supervision, Investigation, Conceptualization. **Yoshihisa Kitayama:** Resources, Investigation, Data curation. **Marie Morooka:** Resources, Investigation. **Tomoki Suichi:** Supervision, Resources, Investigation, Data curation. **Kazumoto Shibuya:** Writing – review & editing, Supervision. **Yoshinori Higuchi:** Writing – review & editing, Supervision, Resources, Project administration, Data curation, Conceptualization. **Satoshi Kuwabara:** Writing – review & editing, Supervision.

## Declaration of competing interest

The authors declare no conflicts of interest.

## Data Availability

The data supporting the findings of this study are available from the corresponding author upon request.
